# Spectral Flow Cytometry Method for Immunophenotyping Neutrophil Activation and NETs in an Acute Dust Exposure Model

**DOI:** 10.1002/iid3.70482

**Published:** 2026-06-30

**Authors:** Logan S. Dean, Maëlis J. L. Wahl, Pinaki Mondal, Tara M. Nordgren, Juwon Park

**Affiliations:** ^1^ Cell and Molecular Biology Graduate Program Colorado State University Fort Collins Colorado USA; ^2^ Department of Environmental and Radiological Health Sciences Colorado State University Fort Collins Colorado USA; ^3^ Department of Biochemistry and Molecular Biology Colorado State University Fort Collins Colorado USA; ^4^ Department of Pediatrics University of Nebraska Medical Center Omaha Nebraska USA; ^5^ Tropical Medicine, Medical Microbiology, and Pharmacology, John A. Burns School Medicine University of Hawai'i at Manoa Honolulu Hawaii USA

**Keywords:** acute lung injury, flow cytometry, NET, neutrophils, organic dust exposure

## Abstract

**Background:**

Although the contributions of neutrophils and neutrophil extracellular trap (NET) in chronic respiratory diseases associated with environmental dust inhalation has advanced, changes in neutrophil populations and their response following acute dust inhalation are relatively limited. Our understanding of neutrophil response during acute lung injury relies on blood and lung samples, which limit investigations on neutrophil dynamics such as neutrophil production, release, trafficking, and NET formation in response to acute exposures.

**Methods:**

To address this limitation, we designed a spectral flow cytometry panel to identify neutrophil progenitors, banded, and mature neutrophils across different sites; bone marrow, blood, lung, and bronchoalveolar lavage fluid (BALF) following acute organic dust extract (ODE) exposure.

**Results:**

We demonstrate that acute ODE exposure increases band and mature neutrophils in the lung and BALF while decreasing band neutrophils in the bone marrow and blood. Interestingly, the proportion of pro‐neutrophils (ProNeu1 and ProNeu2) was altered following ODE in the blood but not bone marrow. We also analyzed the expression of surface markers associated with neutrophil recruitment and activation, as well as their size and granularity after ODE exposure. Regardless of their maturation status, neutrophils in BALF and lung exhibited significant changes in CD11b, CXCR2, and CXCR4 levels post‐ODE, while CD62L levels were specifically elevated in the blood. Finally, we identified lytic and vital NET forming neutrophils via Hoechst 33342 intensity in the lung and BALF, demonstrating increases in NET‐forming neutrophil counts following ODE.

**Conclusion:**

Our spectral flow cytometry method provides valuable insight into neutrophil response, activation, and NET‐forming capacity in response to acute ODE exposure.

## 1 Introduction

Neutrophils, the most abundant leukocytes in human blood, play an essential role in the initial response to acute infection and tissue damage against external insults [1]. They rapidly deploy effector functions, such as degranulation, phagocytosis, and release of neutrophil extracellular traps (NET) to defend the host against invading pathogens and environmental insults [2, 3]. Neutrophil activation is a critical step for resolving inflammatory responses and maintaining homeostasis [4]. However, excessive neutrophil infiltration and activation during initial inflammatory response can lead to granule release and persistent NET formation, perpetuating inflammatory responses, and causing additional tissue injury and/or impairing tissue repair [5, 6].

Elevated NETs in circulation and within tissue have been observed in various chronic lung diseases, including asthma and chronic obstructive pulmonary disease (COPD) and infectious diseases, such as COVID‐19 and streptococcal pneumonia [7, 8, 9]. Studies have demonstrated that host inflammatory factors are involved in triggering NETosis and/or that certain neutrophil subsets are more prone to forming NETs [10, 11]. Most often, NET formation triggers lytic cell death, but it can also proceed without lytic death, termed “vital NET formation”, that is non‐lytic and maintains neutrophils' ability to move and phagocytose following NET release [12]. Accumulating evidence has demonstrated that both vital and lytic NETs have the potential to exacerbate inflammatory pathology and coagulopathy [6, 13, 14, 15, 16, 17].

It is well demonstrated that neutrophilia is a major histopathologic response in the airway and lung tissue to acute exposure of dust extracts [13, 14] and chronic dust exposures are linked to development of chronic lung diseases [14, 15, 16, 17, 18, 19]. Still, neutrophil response, such as changes in neutrophil populations, phenotypes, and functionalities in response to acute insults are less understood, particularly, at mucosal barrier sites, such as the lung [20, 21]. Therefore, it is essential to understand temporal dynamics of neutrophil regulation and response during the acute phase of inflammatory response [22, 23, 24]. In addition, understanding how acute stimuli regulate NET formation, and whether NETosis is lytic, vital, or both in response to such stimuli remains an area of active investigation [22, 23, 24, 25, 26].

Using an established mouse model of acute ODE, we have previously established that a single intranasal challenge with ODE leads to evidence of NETosis in the airways at 5 h following exposure [13]. These findings were limited by using a method that relies on cytocentrifugation of collected airway immune cell populations from bronchoalveolar lavage fluid and semi‐quantitative scoring of “shooting star” morphologies on the slides [13]. This method was limited in its ability to accurately quantify neutrophils that are undergoing NETosis, a lack of ability to differentiate lytic vs vital NETosis and finally presents a major challenge in identifying these cells from alternative samples, such as blood, tissue single cell suspensions, or bone marrow. A comprehensive overview of current methods for NET detection has been extensively reviewed elsewhere [25], but in vitro measurement of NET forming cells is reliant on flow cytometry as the gold standard [26, 27, 28, 29, 30, 31]. A comprehensive flow cytometry‐based panel that incorporates neutrophil progenitors, NET‐forming capacity, and differentiation of lytic vs vital NETosis across relevant tissues in response to an acute inflammatory stimulus, such as ODE, is lacking. To address this limitation, we developed a spectral flow cytometry panel that identifies neutrophil progenitors and banded and mature neutrophils in the bone marrow, blood, lung, and airway in response to acute ODE. Our method allows for characterization of neutrophil phenotypes at the sites ODE, with the ability to examine NET‐forming capacity and identify lytic/non‐lytic NET‐forming signatures via inclusion of the DNA dye Hoechst 33342 [32]. Collectively, this panel combines several aspects of a much‐needed flow cytometry assay for neutrophil and NET formation processes in vivo using a model of acute inflammatory insult in the form of ODE.

## 2 Methods

### 2.1 Preparation of Organic Dust Extracts

Dusts were collected as previously described from swine confinement facilities in the Midwest, United States, and stored at ‐20°C until preparation. Aqueous dust extracts were prepared as previously described [33]; in brief, 5 g of dust was mixed into 50 mL of Hank's Balanced Salt Solution (HBSS) (HyClone, Logan, UT) at room temperature for 1 h. The resulting extract was centrifuged at 2500 × g for 20 min at 4°C. Supernate was transferred to a new 50 mL conical, recentrifuged at 2500 × g for 20 min at 4°C, and subsequent supernate sterile‐filtered with a 0.22 μm filter to produce 100% organic dust extract (ODE). Aliquots of ODE were stored at −20°C until use. Formulations of 12.5% ODE were prepared for animal installations by diluting 100% ODE with phosphate buffered saline (PBS) (Fisher Scientific, Waltham, MA). A dose of 12.5% ODE has been previously demonstrated as appropriate for generating substantial lung inflammation characterized by airway neutrophilia with a single instillation and promoting histopathological markers of lung disease with repetitive exposure, while not leading to significant weight loss, lethargy, or other moribund phenotypes [34, 35, 36, 37, 38].

### 2.2 Animal Husbandry and Installations

Animal protocols were reviewed and approved by the Institutional Animal Care and Use Committee at Colorado State University (Protocol #2887). 7^−^month‐old C57BL/6 male and female mice (Jackson Labs, Bar Harbor, ME) were used. Mice were allowed ad libitum food and water. For intranasal installations, mice were lightly anesthetized under 1.6%–1.8% isoflurane and received a single 50 μL dose of 12.5% ODE or PBS for the control group. Euthanasia of animals was performed with 100% isoflurane overdose followed by cervical dislocation. All animals were used for the experiment, with none excluded. Mice were randomized by cage to determine PBS or ODE exposure. Exposures were given spaced so that euthanasia occurred at 5 h post exposure for each mouse. Exposures received for each mouse were marked on the mouse by the doser and individual responsible for euthanasia.

### 2.3 Sample Extraction and Processing

Following euthanasia, an incision was made through the abdominal wall up to the diaphragm. The diaphragm was punctured and cleared away to allow the lungs to retract fully into the thoracic cavity. Another incision was made to remove the sternum and a cardiac puncture with a 23‐gauge needle was used to collect approximately 500 μL of blood that was placed in K2EDTA Microtainer tubes (BD Biosciences, Franklin Lakes, NJ). Bronchoalveolar lavage fluid (BALF) was collected via three 1 mL lavages using ice cold HBSS (Cytiva, Marlborough, MA) with 0.25% of NaN_3_. Left and right lungs were removed and placed in metal lysing matrix tubes (MP Biomedical, Santa Anna, CA) in 1 mL of HBSS + 0.25% NaN_3_ on ice. Lungs were gently mechanically dissociated utilizing a Bead Mill 24 (Fisherbrand, Waltham, MA) at 2.10 speed for 10 cycles of 15 s with 3 s rest between cycles. Resulting tissues were passed through a 70 µm filter with an additional rinse of 1 mL HBSS + 0.25% NaN_3_ to form a single cell suspension (SCS). To isolate bone marrow cells, femurs and tibias were placed in a 0.65 mL tube nested in a 1.5 mL Eppendorf tube and extracted via centrifugation at 12,000 × g for 30 s for cell collection. Bone marrow cell pellets were resuspended in HBSS + 0.25% NaN_3_ to form a bone marrow SCS. Lung and bone marrow SCS, as well as BALF were centrifuged at 400 × g at 4°C for 8 min, the supernate aspirated off, and resulting cell pellets utilized for flow cytometry staining.

### 2.4 Flow Cytometry‐Mediated Analysis of Neutrophils in BALF, Lung SCS, Bone Marrow SCS, and Blood

The flow cytometry panel was constructed using several recently published manuscripts on NET formation, neutrophil progenitor markers, and analysis of these populations across a range of tissues [29, 32, 39, 40]. All staining was performed protected from light. BALF, lung SCS, bone marrow SCS, and whole blood cell pellets were incubated for 30 min in Ghost Dye Red 780 (Tonbo Biosciences, San Diego CACA, 1:5000) at 4°C in a 96‐well round bottom plate (Fisher Scientific, Waltham, MA). Two‐hundred μL of flow buffer (HBSS + 1% BSA, 0.25% NaN_3_) was added and the cells were centrifuged at 400 × g for 8 min. Resulting supernate was decanted and cells were resuspended in 50 μL of TruStain FcX (BioLegend, San Diego, CA, 1:200) for 15 min at room temperature. Fifty μL of the extracellular antibody cocktail (Supporting Table 1) including Hoechst 33342 was then added and incubated for 30 min at room temperature. Cells were washed and resuspended in BD Cytofix/Cytoperm (BD Biosciences, Franklin Lakes, NJ) for 30 min at 4°C. Cells were washed in BD Perm/Wash (BD Biosciences, Franklin Lakes, NJ) and then resuspended in an intracellular primary antibody cocktail (Supporting Table 1) for 30 min. Cells were washed with BD Perm/Wash and then resuspended in the intracellular secondary antibody cocktail for 30 min. A final wash was performed and then cells were resuspended in flow buffer for analysis. Cells were acquired on a 4‐laser (Violet 405 nm, Blue 488 nm, Yellow‐Green 561 nm, and Red 640 nm) Cytek Auorora Flow Cytometer (Cytek Biosciences, Freemont, CA). Single color compensation beads were utilized for spectral unmixing of fluorescent parameters and fluorescence minus ones (FMOs) were prepared for definitive gating placement (Supporting Figure 4). The resulting data were exported and analyzed using FlowJo Version 10 (Treestar, Ashland, OR) software. Given the large number of events collected for bone marrow (averaged greater than 7 million events), bone marrow FCS files were pre‐gated on “cells” then down sampled into 2 million events for each FCS file before final analysis.

### 2.5 Immunofluorescence Imaging and Quantification

8‐week‐old female and male C57BL/6 mice (Jackson laboratories, Bar Harbor, ME. Stock number: 000664) were randomized to PBS or ODE instillations and sacrificed 5 h post instillations. Lungs were inflated with 10% neutral buffered formalin at a pressure of 20 cmH_2_O for 24 h for fixation and preservation of pulmonary architecture, and subsequently paraffin embedded and sectioned at 5 µm thickness. Sections were baked at 60°C for 1 h, then graded to water through Xylene and alcohol. Antigen retrieval was performed at 95°C for 8 min in a pressure cooker submerged in Tris‐EDTA buffer (pH 9.0). Sections were incubated in TruStain FcX™ (BioLegend, San Diego, CA. Cat. No. 101320) at room temperature for 30 min, then blocked in 10% donkey serum with 1% BSA in PBS with 0.02% Triton X‐100 at room temperature for 1 h. CitH3 (Abcam, Waltham, MA. Cat. No. ab281584) and MPO (R&D, Minneapolis, MN. Cat. No. AF3667) were diluted to 1:250 and 1:100 in the 0.5% blocking solution, respectively, and incubated at 4°C overnight. Fluorescent conjugated secondary antibodies (Thermo Fisher Scientific, Waltham, MA) were applied at 1:500 dilution in 0.5% blocking solution for 1 h at room temperature. The sections were counterstained with DAPI and mounted (Vector Labs, Newark, CA. Cat. No. H‐1200‐10). Images were acquired on a Zeiss 800 CLSM at 200X magnification and quantified by QuPath.

### 2.6 Statistics

GraphPad Prism software (Version 10, La Jolla, CA) was utilized to perform ROUT outlier analysis (Q = 1%), with resulting data analyzed via a Mann‐Whitney *U* test for between‐groups comparisons if non‐normally distributed. If data were normally distributed, *t*‐tests were utilized for between‐group comparisons. Differences between groups were considered significant if the *p* value ≤ 0.05. Data are represented with median ± 95% confidence interval on all figures unless otherwise noted. Power analysis was conducted to determine appropriate number of animals per sex per exposure condition.

## 3 Results

### 3.1 Total Band and Mature Neutrophils Increase in the Airway and Lung Following Acute Organic Dust Extract Exposure

**Figure 1:**
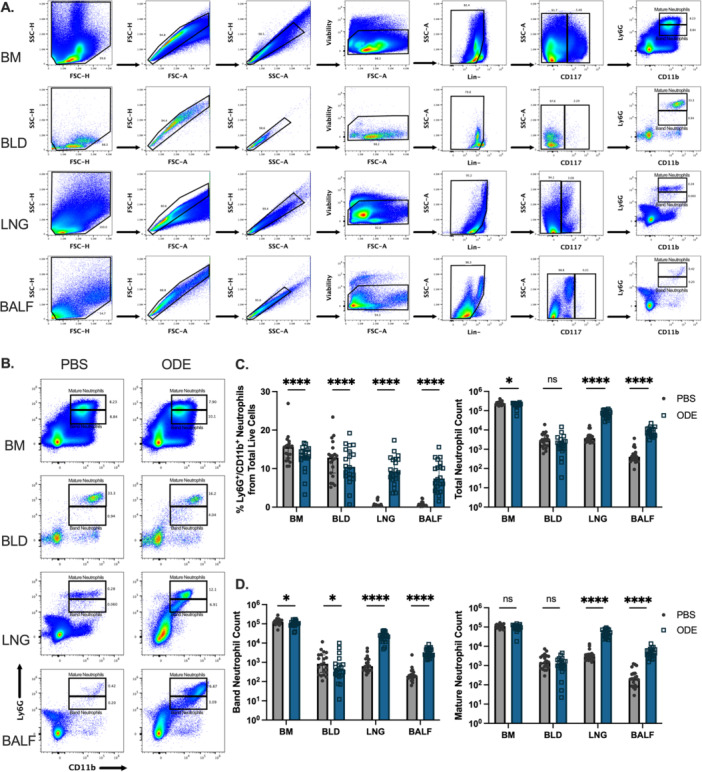
(A) Representative flowcytometry gating strategy of PBS‐exposed mice bone marrow (BM), blood (BLD), lung (LNG), and bronchoalveolar lavage (BALF) for total neutrophils (Live/Lin^−^/CD117^−^/Ly6G^+^ /CD11b^+^ ). (B) Comparison of PBS‐ and ODE‐exposed samples representative gating strategies for identification of total neutrophils and identification of band (Live/Lin^−^/CD117^−^/Ly6G^Lo^/CD11b^Lo^) and mature (Live/Lin^−^/CD117^−^/Ly6G^Hi^/CD11b^Hi^) neutrophils in BM, BLD, LNG, and BALF. (C) Percentage of total neutrophils from live cells in BM, BLD, LNG, and BALF in PBS and ODE‐exposed mice. Quantification of total neutrophils in BM, BLD, LNG, and BALF in PBS‐ and ODE‐exposed mice. (D) Quantification of band and mature neutrophils in BM, BLD, LNG, and BALF in PBS‐ and ODE‐exposed mice. Each dot represents a single animal. PBS (*n* = 20) and dust (*n* = 20) exposed mice were 50:50 male:female. Bars are median with 95% confidence interval. Mann Whitney‐*U* Test. *p < 0.05, ****p < 0.0001, ns = non‐significant.

To better understand the acute inflammatory response of neutrophils to inhaled dust, we utilized spectral flow cytometry to characterize and compare neutrophil populations (neutrophil progenitor, band, and mature neutrophils) in the bone marrow, circulation and airways under steady state and acute dust extract‐exposed conditions. To assess changes in neutrophil populations after acute exposure of dust, we chose organic dust extracts (ODE) based on our previous observation, which showed neutrophilia in the airway as a notable pathologic feature in mice 5 h after a single intranasal ODE instillation [36, 41, 42]. We gave a single intranasal instillation to mice of 12.5% ODE or PBS as a control group and collected bone marrow (BM), blood (BLD), lung (LNG), and bronchoalveolar lavage fluid (BALF) samples 5 h post‐instillation. Firstly, samples from PBS‐instilled (control) mice were used to establish and optimize flow gating to identify neutrophils in four different locations, such as BM, BLD, LNG, and BALF. We used makers for non‐neutrophil lineage (CD3, CD19, B220, Ter119 hereafter referred to as Lin) and hematopoietic stem cells (CD117/c‐Kit) to eliminate them prior to gating myeloid cells. Representative gating plots demonstrated band (Lin^−^/CD117^−^/CD11b^+^/Ly6G^Lo^) and mature neutrophils (Lin^−^/CD117^−^/CD11b^+^/Ly6G^Hi^) in the different locations based on the gating strategy in Figure 1A. To validate the gating strategy to identify band and mature neutrophils, we analyzed median fluorescence intensity (MFI) of CXCR2 and CXCR4 in each population. CXCR2 expression was significantly higher in mature neutrophils, compared to band neutrophils, but CXCR4 expression was comparable (Supporting Figure 1). The ODE group showed increased neutrophil populations in BLD and BALF, but not in BM and LNG compared to PBS control group (Figure 1B). Further quantification of neutrophil proportions and counts demonstrated that the ODE group had significantly decreased proportions of total neutrophils (CD11b^+^/Ly6G^+^) in the BM and BLD while the proportion significantly increased in LNG and BALF, compared to PBS controls (Figure 1C). We also observed a similar trend with increased total neutrophil counts in LNG and BALF in ODE groups, but decreased neutrophil counts in BM. However, BLD neutrophil counts were comparable between groups (Figure 1C). When split into band (Ly6G^Lo^) and mature (Ly6G^Hi^) neutrophils based on Ly6G expression [43], total band neutrophils decreased in BM and BLD but drastically increased in LNG and BALF of ODE mice compared to control mice (Figure 1D). ODE exposure significantly increased mature neutrophil populations, but there were no changes in these populations in the BM or BLD (Figure 1D), suggesting that circulating neutrophils are rapidly recruited to the bronchoalveolar space and lung tissue in response to acute ODE.

### 3.2 The Proportion of Neutrophil Precursors Are Altered in Blood but Not Bone Marrow of ODE‐Exposed Mice

**Figure 2:**
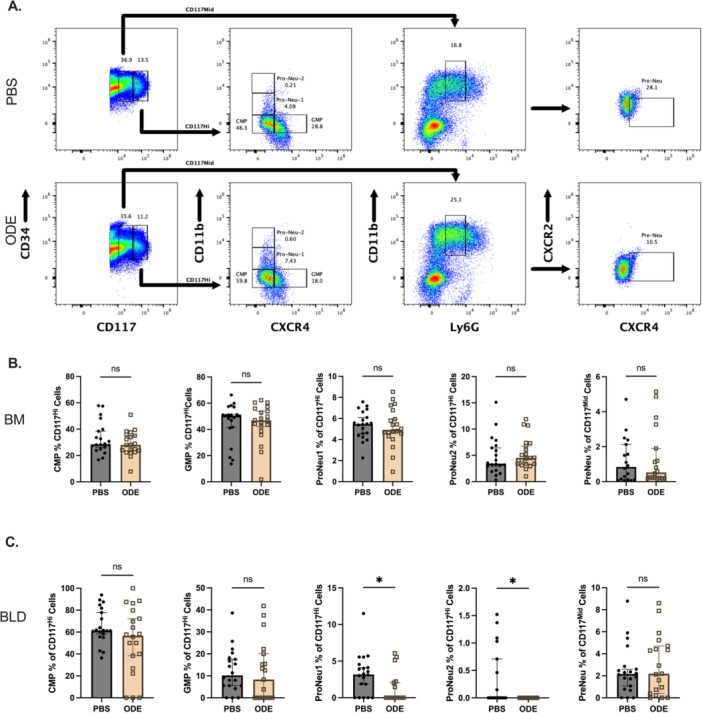
(A) Representative gating strategy of neutrophil precursor identification in bone marrow of PBS‐ and ODE‐exposed mice. (B) Proportions of common myeloid progenitors (CMP), granulocyte myeloid progenitor (GMP), ProNeu1, ProNeu2, and PreNeu in PBS‐ and ‐ODE‐exposed mice bone marrow. (C) Proportions of common myeloid progenitors (CMP), granulocyte myeloid progenitor (GMP), ProNeu1, ProNeu2, and PreNeu in PBS‐ and ODE‐exposed mice blood. PBS‐ (*n* = 20) and ODE‐exposed (*n* = 20) mice were 50:50 male:female. median with 95% confidence interval. Mann Whitney‐*U* Test. *p < 0.05, ns = non‐significant.

To assess the effect of acute ‐ODE exposure on neutrophil development, we conducted a detailed analysis of neutrophil progenitors and precursors in the BM and BLD. To identify neutrophil progenitors in the BM, we analyzed the expression of CD117, CD11b, CXCR4, and Ly6G in Lin‐negative cells by flow cytometry. We identified common myeloid progenitor (CMP, Lin^−^/CD117^Hi^/CXCR4^+^/CD11b^−^), granulocyte‐monocyte progenitors (GMP, Lin^−^/CD117^Hi^/CXCR4^−^/CD11b^−^), Pro‐Neu1 (Lin^−^/CD117^Hi^, CXCR4^−^/CD11b^Lo^), ProNeu2 (Lin^−^/CD117^Hi^/CXCR4^−^/CD11b^Hi^), and Pre‐Neu (Lin^−^/CD117^Mid^/CXCR4^+^/CXCR2^−^) cells as neutrophil precursors in the BM of PBS and ODE‐exposed mice (Figure 2A). There were no changes in the proportion of neutrophil progenitors and precursors in BM between groups (Figure 2B). Interestingly, in circulation, CMP, GMP, and Pre‐Neu proportions remained unchanged, but reductions in ProNeu1 and ProNeu2 proportions were observed in ODE mice (Figure 2C). These observations in ODE mice may explain the decreased band neutrophil counts in BLD.

### 3.3 Band and Mature Neutrophil Phenotypes Are Altered in Response to ODE

**Figure 3:**
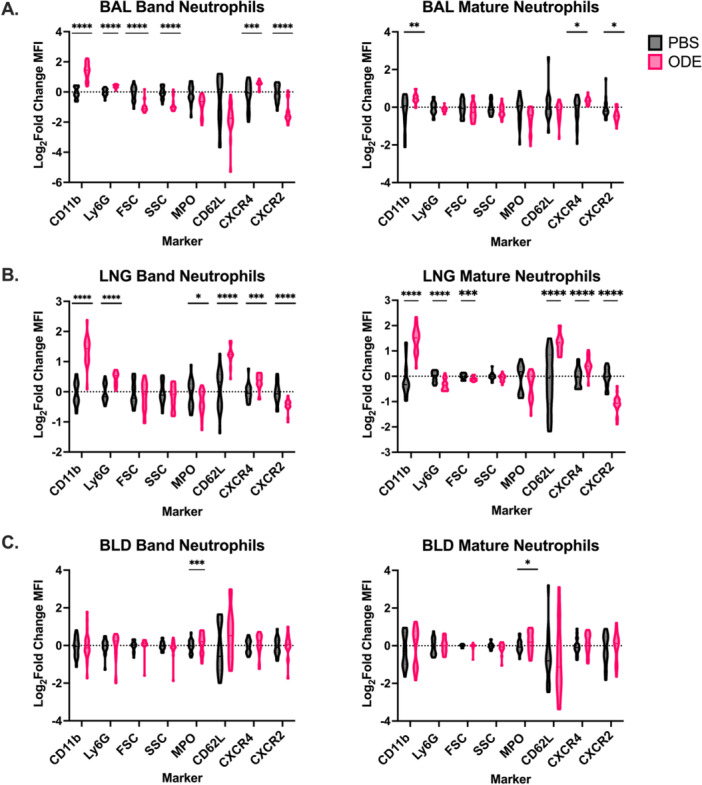
Log2 fold change of median fluorescent intensity (MFIs) of band and mature neutrophils in BALF (A) lung (B) and blood (C) of PBS‐ and ODE‐exposed animals. PBS (*n* = 20) and ‐ODE (*n* = 20) exposed mice were 50:50 male:female. Line is at mean. *t*‐Test *p < 0.05, **p < 0.01, ***p < 0.001, ****p < 0.0001, no comparison shown equals non‐significant.

Next, to understand the activation state and phenotype of neutrophils at sites of neutrophil migration from blood to lung following acute ODE, we determined the expression of neutrophil‐related markers (CD11b, CXCR4, CXCR2, Ly6G, CD62L, myeloperoxidase [MPO]) on band and mature neutrophils in BALF, LNG, and BLD. Neutrophil size and granularity can be indicative of functionality and activity, thus we also analyzed MFI of forward scatter (FSC) and side scatter (SSC) [40, 44]. When we analyzed MFI of indicated markers, band neutrophils in BALF and LNG showed increases in CD11b, Ly6G, and CXCR4 MFI in response to ODE (Figure 3A,B). BALF band neutrophils decreased FSC and SSC MFI as well as CXCR2 in response to ODE (Figure 3A). Similarly, mature neutrophils in BALF and LNG increased CD11b and CXCR4, but decreased CXCR2 and MPO MFIs (Figure 3A,B). LNG from ODE mice showed a markedly increased MFI of CD62L in mature and band neutrophils but exhibited no change in BALF (Figure 3AB). LNG band neutrophils showed a significant increase in Ly6G MFI, while Ly6G and FSC MFI were decreased in mature neutrophils (Figure 3B). To determine if these phenotypic changes also occurred systemically, not just at the site of local inflammation, we further examined MFI of markers in BLD band and mature neutrophils (Figure 3C). Only MPO MFI was statistically increased in both BLD band and mature neutrophils, but other marker MFIs were comparable between groups (Figure 3C). These data suggest that acute ODE exposure alters the expression of markers associated with neutrophil integrin‐mediated movement (CD11b, Ly6G, and CD62L), chemotactic gradient response (CXCR4 and CXCR2), and morphologic changes (FSC and SSC) in band and mature neutrophils within the lower respiratory system.

### 3.4 NET‐Forming Band and Mature Neutrophils Increase in BALF and Lung Following Acute ODE Exposure

**Figure 4:**
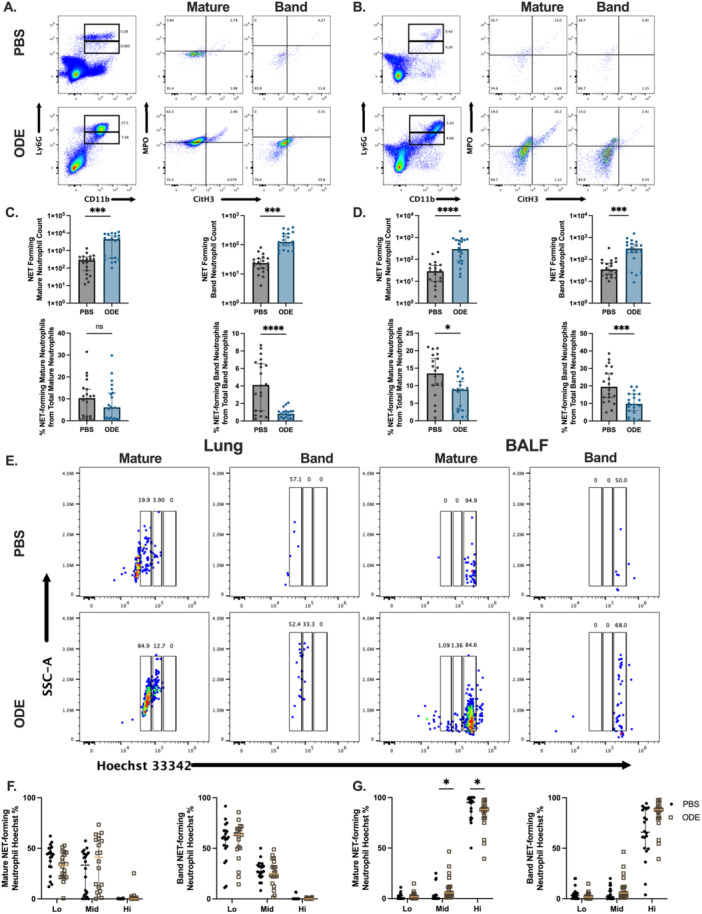
Representative gating strategy for identifying NET‐forming neutrophils from parent band and mature neutrophil populations in the lung (A) and BALF (B) in PBS‐ and ODE‐exposed mice. Quantification of NET forming neutrophils in mature and band neutrophils in lung (C) and BALF (D) in PBS‐ and ODE‐exposed mice. (E) Representative gating strategy for NET forming mature and band neutrophils from lung and BALF in PBS‐ and ODE‐exposed mice. Proportions of mature and band NET forming neutrophils in lung (F) and BALF (G) PBS‐ (*n* = 20) and ODE‐exposed (*n* = 20) mice were 50:50 male:female. Samples on the left‐most half of graphs/images are from lung and samples on the right‐most half of graphs/images are from BALF. Line and bars are at the median with 95% confidence interval. Mann Whitney‐*U* Test *p < 0.05, ***p < 0.001, ****p < 0.0001, no comparison shown equals non‐significant.

Previously, NET‐forming cells were detected in BALF cells from single‐ODE instillation mice [45], providing evidence of NET formation in the airway. We assessed the presence of NETs in ODE‐instilled lungs by immunostaining for MPO and CitH3. ODE mice showed a significant increase in NET forming (MPO^+^CitH3^+^) cells within the lung tissue (Supporting Figure 2A,B), compared to PBS mice. Next, to examine how acute ODE exposure impacts neutrophils to form NETs, we included MPO and CitH3 antibodies in our flow panel and performed fluorescence minus one (FMO) controls for each antibodies to ensure appropriate gating to identify NET‐forming neutrophils (Supporting Figure 4). We analyzed NET‐forming neutrophils (MPO^+^ CitH3^+^) in band and mature neutrophils from LNG (Figure 4A) and BALF samples (Figure 4B). ODE mice significantly increased the number of NET‐forming neutrophils in LNG and BALF samples, regardless of neutrophil maturation status (Figure 4C,D). Interestingly, the proportion of NET‐forming neutrophils was higher in PBS controls than in acute ODE samples (Figure 4C,D). These data suggest that the increased count of NET forming cells in ODE group is reflective greater numbers of neutrophils present, rather than an increased capacity to form NETs. As the NETosis process can be lytic (full rupture of the neutrophil membrane) and/or non‐lytic (maintaining membrane integrity), we utilized Hoechst 33342 signal intensity as a method to examine changes indicative of DNA presence or release of DNA from cells as previously described (Figure 4E) [32, 46, 47, 48, 49, 50]. Hoechst^Hi^ indicates a more vital‐like NET, Hoechst^Lo^ indicates a more lytic NET, and Hoechst^Mid^ is in between lytic and vital NET‐release patterns. As the pre‐processing gate uses a viability dye, where higher signal intensity equals non‐live cells, these patterns of Hoechst intensity indicate NET‐forming cells, not NET‐released (cytoplast‐like) cells. In the LNG, both band and mature neutrophil populations, regardless of ODE exposure, exhibited primarily Hoechst^Lo^ and Hoechst^Mid^ NET‐forming populations (Figure 4F). In BALF samples, the Hoechst^Hi^ populations dominated in NET‐forming band and mature neutrophils, with a significant increase in Hoechst^Hi^ cells in band neutrophils from the ODE group (Figure 4G). Furthermore, we observed increased MPO^+^ band and mature neutrophils in the lungs following ODE exposure (Supporting Figure 3A,B). This coincided with an increased proportion of MPO^+^ band, but not mature neutrophils (Supporting Figure 3C,D). Collectively, these data demonstrate that acute ODE exposure increases activated neutrophils, both NET‐forming and MPO^+^ neutrophils likely due to increased neutrophil counts due to inflammation, and that the lytic state of these neutrophils differs between BALF and LNG samples.

## 4 Discussion

Although the understanding of neutrophil diversity in chronic lung diseases is increasing and evolving, characteristics of neutrophils and their phenotypic and functional alterations during the early phase of inflammatory responses remain under active investigation. In this study, we aimed to gain more insight into changes in neutrophil populations and phenotype from neutrophil development to migration (bone marrow and blood) and at the site of acute inflammation (airway and lung tissue). Using a model of acute inhalational ODE‐induced airway neutrophilia, we applied a spectral flow cytometry methodological framework to identify neutrophil progenitors, band and mature neutrophils, and identify changes in cellular phenotype within circulation and the lung following acute lung injury. Besides conventional flow cytometry for use in immunophenotyping, spectral flow cytometry presents increased panel resolution and marker selection due to the high sensitivity of detection, allowing more detailed characterization of cell types of interest. We demonstrate that neutrophil populations exhibit phenotypic alterations in both band and mature neutrophils in a location‐dependent manner after acute ODE. More importantly, we validated a reliable method of identifying and quantifying lytic and non‐lytic NET‐forming neutrophils by flow cytometry analysis at the site of local inflammatory processes following exposure to acute ODE. Our data demonstrates that both mature and band neutrophils form NETs, and that NET‐forming cell counts were significantly increased in BALF and LNG 5 h post‐instillation of ODE compared to PBS vehicle‐instilled mice.

Interestingly, the acute ODE instillation did not affect the capacity of neutrophils to form NETs, but this increase was due to the total increase of neutrophils following ODE for all but mature neutrophils within the LNG. Of note, we observed that the propensity for neutrophils to undergo lytic versus non‐lytic NET differed between BALF and LNG, regardless of exposure. Collectively, with a spectral flow cytometry‐based analysis of neutrophils, this study provides additional insights into neutrophil dynamics and phenotypic changes during acute inflammation in the context of dust exposure.

Corroborated with the observed neutrophilia in mice 5 h post‐ODE instillation, we found increased numbers of band and mature neutrophils in LNG and BALF [13, 36]. This observation suggests that neutrophils extravasate sufficiently to the lung tissue and bronchoalveolar space by 5 h after ODE challenge. Interestingly, the decreased numbers of neutrophils in BM and BLD in response to ODE were observed only in band neutrophils, but not mature neutrophils. This suggests that acute ODE promotes recruitment of circulating mature neutrophils and likely promotes maturation of recruited band neutrophils in the lung and bronchoalveolar space rather than stimulating robust neutrophil production in the bone marrow. In an acute lung injury model utilizing LPS, neutrophil populations in the blood and lung, as well as bone marrow, followed similar kinetics at 6 h post LPS administration as our 5 h post instillation timepoint [51]. Intriguingly, they demonstrated that GM‐CSF levels peaked at 3 h in the BALF samples, and its cognate receptor GM‐CSFRα remained elevated up to 6 h post LPS on recruited neutrophils, with GM‐CSFRβ increasing expression at 6 and 24 h post LPS. These timepoints for neutrophil dynamics have been further corroborated in human challenge models with similar results and findings at the same 3‐ and 6‐h timepoints, regardless of dose [52]. Another likely candidate for this potential maturation response in the lung is G‐CSF. In the context of acute viral and bacterial infections within the respiratory tract, targeting G‐CSF to reduce neutrophilia has been proven successful, providing a potential means for therapeutic intervention in ODE‐related disease [53].

Different capabilities of band and mature neutrophils to produce NETs have been reported, with band neutrophils traditionally exhibiting decreased ability and frequency of NET formation compared to their mature forms [54, 55, 56]. Our data support the idea of niche‐specific forms of NET formation, challenging the paradigm that band and mature neutrophils form NETs with different capacities. We observed lytic NET formation (characterized by Hoechst^Low^ cells) within LNG neutrophils regardless of maturity and ODE. In BALF samples, the proportion of Hoechst^Mid^ (representing an intermediate state between lytic and vital NET) was increased, but Hoechst^High^ (vital NETs) was reduced in mature neutrophils following ODE exposure. These data suggest that BALF cells predominantly exhibit vital NET formation and appear to undergo lytic NET formation following ODE exposure. However, NETs were rarely formed vital NETs in the lungs from ODE mice. As mature neutrophils have canonically been associated with NET formation [57, 58, 59], our findings provide evidence that acute inflammatory environments induced by ODE within the bronchoalveolar space and lung tissue of the respiratory system may alter NET formation capability. This is particularly important, as non‐lytic NET formation produces cytoplasts that have been demonstrated to be potent inducers of other inflammatory‐based respiratory diseases such as severe asthma [60]. The ability to distinguish between these forms of NET formation provides critical insight into how neutrophil cytoplasts after non‐lytic NET release contribute to exacerbating the inflammatory environment present during more repetitive forms of ODE.

Our investigation into changes in neutrophil phenotype in response to acute dust exposure in the LNG, BALF, and BLD with analysis of MFIs of several markers associated with neutrophil activation yielded some interesting findings. The finding of increased MPO MFI in BLD neutrophils could be indicative of MPO release by neutrophils at the site of ODE, and a potential priming effect on neutrophils found within the BLD post ODE. In a previous study with single ODE challenge, MPO levels in BALF were found to be significantly elevated, supporting this hypothesis [13]. There was also a significant increase in lung mature and band neutrophils expression of CD62L following dust exposure. This markers' reduced expression (CD62L^Lo^) has been traditionally utilized in conjunction with CXCR4^Hi^ expression to denote an aged neutrophil phenotype, and CD62L^Hi^ expression to denote a younger phenotype [61]. In the context of lung fibrosis, these aged neutrophils (denoted by CD62L^Lo^ CXCR4^Hi^) exhibit increased lung retention and an increase in inflammatory phenotype, neutrophil elastase production, and NET formation [62]. CD62L^Lo^ neutrophils within circulation are predictive for COPD disease severity and are associated with obstructive airway disease presence [63]. In healthy BALF and blood, CD62L expression has been proposed as a marker of activation of neutrophils recruited/extravasated into the lung, independent of inflammation [64]. When CD62L expression decreases (some studies demonstrate that it is shed extracellularly and measurable in BALF) from lung neutrophils, this is indicative of an aging phenotype [63, 65]. Given that aging neutrophils exhibit an increased inflammatory profile and therefore propensity for exacerbating inflammatory lung disease, the ODE‐induced increase in CD62L MFI within LNG, and reduction in BALF indicates that acute ODE may cause tissue‐dependent modulation of this marker [66].

Previous methods to quantify NET‐forming neutrophils in response to ODE have relied on the use of cytospins of BALF‐derived cells followed by a differential staining and quantitative scoring of NET presence [13]. Although useful, our flow cytometry method represents an advancement compared to the cytology‐based NET‐forming neutrophil identification in that it allows for the characterization/phenotyping of cells and extends the ability to identify NETs to tissue samples. Within the lung, current published flow cytometry methods of analyzing NET‐forming neutrophils utilize DAPI and SytoxOrange, which only measures release of DNA from neutrophils as a proxy for NET formation [28]. Via the use of CitH3 and MPO as a dual positive marker, along with a viability dye and a DNA binding dye (Hoechst 33342), we ensure that NET formation identification has the molecular markers known to facilitate DNA decondensation and membrane disruption (CitH3 and MPO, respectively) [32]. The multiparametric advantage of spectral flow cytometry allows for characterization of the phenotype of NET‐forming neutrophils, allowing further identification of which neutrophil population is most involved in NET generation, increasing the specificity of further investigations interested in understanding NETs' contributions to inflammation and resolution processes [27].

Our study has several limitations, the first of which is that our identification of NET‐forming neutrophils proceeds through a live cell gate via the use of a fixable viability dye. Given that the majority of what is known of NET formation is based on the lytic form of NETosis, our identification of NET‐forming neutrophils relies on the identification of both MPO and CitH3, within a live cell (membrane intact) population. This identification strategy is meant to prevent non‐specific binding of antibodies used for flow cytometry identification but does prevent the identification of NET‐formed (those that have already released their NET) neutrophils. Secondly, the reagents available for CitH3 and MPO identification within murine samples are limited. Our strategy relies on a primary and fluorophore conjugated secondary for CitH3 and MPO identification, and secondary donkey‐anti‐goat and goat‐anti‐rabbit antibodies, possibly contributing to non‐specific binding of these secondaries. To ensure appropriate gating, FMO's can be used or staining can be done sequentially for the secondary antibodies. Thirdly, this study used a model of acute dust exposure, a known inducer of neutrophil airway recruitment, to examine how ODE alters maturation and activation of neutrophils following acute exposure. This acute timepoint only captures a snapshot of neutrophil response and recruitment, and future studies should examine the impact of repetitive dust exposure models and how they alter NET formation, as well as how recovery from repetitive ODE is mediated by neutrophils and their NET formation. Tertiarily, advances in the use and availability of imaging cytometers have the capacity to provide visual confirmations of populations not feasible by traditional flow cytometry approaches [67]. These methodological advances have great potential for enhancing NET formation studies but require continued implementation and optimization for widespread utility. How these neutrophil dynamics play out in repetitive exposure and recovery following ODE would offer translatable insights into the role of neutrophils in airway disease within workers repetitively exposed in agricultural and livestock occupations.

## Author Contributions

**Logan S. Dean:** investigation, writing – original draft, methodology, validation, visualization, writing – review and editing, formal analysis, data curation, conceptualization. **Maëlis J. L. Wahl:** methodology, writing – review and editing, data curation. **Pinaki Mondal:** investigation, methodology, writing – review and editing, conceptualization. **Tara M. Nordgren:** conceptualization, funding acquisition, writing – review and editing, project administration, resources, supervision. **Juwon Park:** conceptualization, funding acquisition, writing – original draft, methodology, writing – review and editing, project administration, resources, supervision.

## Conflicts of Interest

The authors declare no conflicts of interest.

## Supporting information


**Figure S1:** Median Fluorescent Intensity (MFI) of CXCR2 and CXCR4 in banded and mature neutrophils in bone marrow from PBS‐expose mice. Mann Whitney‐*U* Test, ****p < 0.0001.

**Figure S2**: Cell expressing CitH3, MPO, or co‐expressing CitH3 and MPO were significantly increased following ODE. (A) Representative images of CitH3 and MPO immune‐fluorescence staining in PBS‐ and ODE‐exposed mice (200X magnification, scale bar 100 µM). (B) Quantification of 5 images from PBS‐ and ODE‐exposed mice (*n* = 4). Each dot represents total cell numbers expressing the proteins from a single image. Asterisks denote a two‐tail *p* value ≤ 0.05 as computed by unpaired Mann Whitney‐*U* test.

**Figure S3:** (A) MPO^+^ band and mature neutrophil counts and (B) percentages in the lung samples exposed to PBS or ODE. Mann Whitney‐*U* Test *p < 0.05, ****p < 0.0001, ns = non‐significant.

**Figure S4**: Representative gating strategy for fluorescent minus one (FMO) samples utilized for gating placement for NET formation markers MPO and CitH3 in the lung and BALF in mature and band neutrophil populations6.

**Table S1:** Fluorescently conjugated antibodies used for flow cytometry. Panel denotes extracellular (ex) and intracellular (in) location of markers.

## Data Availability

The data that support the findings of this study are available from the corresponding author upon reasonable request.

## References

[1] C. Nathan , “Neutrophils and Immunity: Challenges and Opportunities,” Nature Reviews Immunology 6 (2006): 173–182. 10.1038/nri1785 16498448

[2] W. M. Nauseef and N. Borregaard , “Neutrophils at Work,” Nature Immunology 15 (2014): 602–611. 24940954 10.1038/ni.2921

[3] C. M. de Bont , N. Eerden , W. C. Boelens , and G. J. M. Pruijn , “Neutrophil Proteases Degrade Autoepitopes of NET‐Associated Proteins,” Clinical & Experimental Immunology 199 (2019): 1–8. 31661552 10.1111/cei.13392 PMC6904661

[4] J. Grommes and O. Soehnlein , “Contribution of Neutrophils to Acute Lung Injury,” Molecular Medicine 17 (2011): 293–307. 21046059 10.2119/molmed.2010.00138 PMC3060975

[5] A. E. Jasper , W. J. McIver , E. Sapey , and G. M. Walton , “Understanding the Role of Neutrophils in Chronic Inflammatory Airway Disease,” F1000Research 8 (2019): 557. 10.12688/f1000research.18411.1. PMC6489989 31069060

[6] S. L. Tucker , D. Sarr , and B. Rada , “Neutrophil Extracellular Traps Are Present in the Airways of ENaC‐Overexpressing Mice With Cystic Fibrosis‐Like Lung Disease,” BMC Immunology 22 (2021): 7. 33478382 10.1186/s12865-021-00397-w PMC7819174

[7] J. Chen , T. Wang , X. Li , et al., “DNA of Neutrophil Extracellular Traps Promote NF‐κB‐dependent Autoimmunity via cGAS/TLR9 in Chronic Obstructive Pulmonary Disease,” Signal Transduction and Targeted Therapy 9 (2024): 163. 38880789 10.1038/s41392-024-01881-6 PMC11180664

[8] B. N. Porto and R. T. Stein , “Neutrophil Extracellular Traps in Pulmonary Diseases: Too Much of a Good Thing?,” Frontiers in Immunology 7 (2016): 311. 10.3389/fimmu.2016.00311. 27574522 PMC4983612

[9] R. D. Gray , “NETs in Pneumonia: Is Just Enough the Right Amount?,” European Respiratory Journal 51 (2018): 1800619. 29700108 10.1183/13993003.00619-2018

[10] F. Nappi , F. Bellomo , and S. S. Avtaar Singh , “Insights into the Role of Neutrophils and Neutrophil Extracellular Traps in Causing Cardiovascular Complications in Patients With COVID‐19: A Systematic Review,” Journal of Clinical Medicine 11 (2022): 2460. 10.3390/jcm11092460. 35566589 PMC9104617

[11] R. Dalan , “Metformin, Neutrophils and COVID‐19 Infection,” Diabetes Research and Clinical Practice 164 (2020): 108230. 32446796 10.1016/j.diabres.2020.108230 PMC7242188

[12] F. H. Pilsczek , D. Salina , K. K. H. Poon , et al., “A Novel Mechanism of Rapid Nuclear Neutrophil Extracellular Trap Formation in Response to *Staphylococcus aureus* ,” Journal of Immunology 185 (2010): 7413–7425. 10.4049/jimmunol.1000675 21098229

[13] E. C. Dominguez , A. J. Heires , J. Pavlik , et al., “A High Docosahexaenoic Acid Diet Alters the Lung Inflammatory Response to Acute Dust Exposure,” Nutrients 12 (2020): 2334. 32759853 10.3390/nu12082334 PMC7468878

[14] T. M. Nordgren and K. L. Bailey , “Pulmonary Health Effects of Agriculture,” Current Opinion in Pulmonary Medicine 22 (2016): 144–149. 26761627 10.1097/MCP.0000000000000247 PMC4764055

[15] T. M. Nordgren and C. Charavaryamath , “Agriculture Occupational Exposures and Factors Affecting Health Effects,” Current Allergy And Asthma Reports 18 (2018): 65. 30291457 10.1007/s11882-018-0820-8 PMC6644660

[16] A. J. Heires , D. Samuelson , D. Villageliu , T. M. Nordgren , and D. J. Romberger , “Agricultural Dust Derived Bacterial Extracellular Vesicle Mediated Inflammation Is Attenuated by DHA,” Scientific Reports 13 (2023): 2767. 36797300 10.1038/s41598-023-29781-9 PMC9933036

[17] J. A. Poole , T. A. Wyatt , S. G. Von Essen , et al., “Repeat Organic Dust Exposure–Induced Monocyte Inflammation Is Associated With Protein Kinase C Activity,” Journal of Allergy and Clinical Immunology 120 (2007): 366–373. 17555806 10.1016/j.jaci.2007.04.033

[18] J. A. Poole , T. M. Nordgren , A. J. Heires , et al., “Amphiregulin Modulates Murine Lung Recovery and Fibroblast Function Following Exposure to Agriculture Organic Dust,” American Journal of Physiology‐Lung Cellular and Molecular Physiology 318 (2020): L180–L191. 31693392 10.1152/ajplung.00039.2019 PMC6985879

[19] K. Warren , T. Wyatt , D. Romberger , et al., “Post‐Injury and Resolution Response to Repetitive Inhalation Exposure to Agricultural Organic Dust in Mice,” Safety 3 (2017): 10. 29387711 10.3390/safety3010010 PMC5788309

[20] A. Jo and D. W. Kim , “Neutrophil Extracellular Traps in Airway Diseases: Pathological Roles and Therapeutic Implications,” International Journal of Molecular Sciences 24 (2023): 5034. 10.3390/ijms24055034. 36902466 PMC10003347

[21] T. Pan and J. W. Lee , “A Crucial Role of Neutrophil Extracellular Traps in Pulmonary Infectious Diseases,” Chinese Medical Journal Pulmonary and Critical Care Medicine 2 (2024): 34–41. 39170960 10.1016/j.pccm.2023.10.004 PMC11332830

[22] R. C. Mettelman , E. K. Allen , and P. G. Thomas , “Mucosal Immune Responses to Infection and Vaccination in the Respiratory Tract,” Immunity 55 (2022): 749–780. 35545027 10.1016/j.immuni.2022.04.013 PMC9087965

[23] R. J. Hewitt and C. M. Lloyd , “Regulation of Immune Responses by the Airway Epithelial Cell Landscape,” Nature Reviews Immunology 21 (2021): 347–362. 10.1038/s41577-020-00477-9 PMC7804588 33442032

[24] T. M. Chamardani and S. Amiritavassoli , “Inhibition of NETosis for Treatment Purposes: Friend or Foe?,” Molecular and Cellular Biochemistry 477 (2022): 673–688. 34993747 10.1007/s11010-021-04315-x PMC8736330

[25] M. Stoimenou , G. Tzoros , P. Skendros , et al., “Methods for the Assessment of NET Formation: From Neutrophil Biology to Translational Research,” International Journal of Molecular Sciences 23 (2022): 15823. 10.3390/ijms232415823. 36555464 PMC9781911

[26] S. Masuda , S. Shimizu , J. Matsuo , et al., “Measurement of NET Formation In Vitro and In Vivo by Flow Cytometry,” Cytometry, Part A 91 (2017): 822–829. 10.1002/cyto.a.23169 PMC5601186 28715618

[27] E. Schneck , F. Mallek , J. Schiederich , et al., “Flow Cytometry‐Based Quantification of Neutrophil Extracellular Traps Shows an Association With Hypercoagulation in Septic Shock and Hypocoagulation in Postsurgical Systemic Inflammation—A Proof‐of‐Concept Study,” Journal of Clinical Medicine 9 (2020): 174. 10.3390/jcm9010174. 31936385 PMC7019434

[28] O. Zharkova , S. H. Tay , H. Y. Lee , et al., “A Flow Cytometry‐Based Assay for High‐Throughput Detection and Quantification of Neutrophil Extracellular Traps in Mixed Cell Populations,” Cytometry, Part A 95 (2019): 268–278. 10.1002/cyto.a.23672 PMC6590256 30549398

[29] C. J. McGill , R. J. Lu , and B. A. Benayoun , “Protocol for Analysis of Mouse Neutrophil NETosis by Flow Cytometry,” STAR Protocols 2 (2021): 100948. 34820637 10.1016/j.xpro.2021.100948 PMC8599168

[30] A. Cossarizza , H. D. Chang , A. Radbruch , et al., “Guidelines for the Use of Flow Cytometry and Cell Sorting in Immunological Studies (Third Edition),” European Journal of Immunology 51 (2021): 2708–3145. 34910301 10.1002/eji.202170126 PMC11115438

[31] E. A. Barbu , V. M. Dominical , L. Mendelsohn , and S. L. Thein , “Detection and Quantification of Histone H4 Citrullination in Early NETosis With Image Flow Cytometry Version 4,” Frontiers in Immunology 11 (2020): 1335. 10.3389/fimmu.2020.01335. 32765493 PMC7378400

[32] Y. P. Zhu , M. Speir , Z. Tan , et al., “NET Formation Is a Default Epigenetic Program Controlled by PAD4 in Apoptotic Neutrophils,” Science Advances 9 (2023): 1397. 10.1126/sciadv.adj1397. PMC10732518 38117877

[33] D. J. Romberger , V. Bodlak , S. G. Von Essen , T. Mathisen , and T. A. Wyatt , “Hog Barn Dust Extract Stimulates IL‐8 and IL‐6 Release in Human Bronchial Epithelial Cells via PKC Activation,” Journal of Applied Physiology 93 (2002): 289–296. 12070216 10.1152/japplphysiol.00815.2001

[34] A. Ulu , J. V. Velazquez , A. Burr , et al., “Sex‐Specific Differences in Resolution of Airway Inflammation in Fat‐1 Transgenic Mice Following Repetitive Agricultural Dust Exposure,” Frontiers in Pharmacology 12 (2022): 785193. 10.3389/fphar.2021.785193. 35095496 PMC8793679

[35] T. M. Nordgren , A. J. Heires , K. L. Bailey , et al., “Docosahexaenoic Acid Enhances Amphiregulin‐Mediated Bronchial Epithelial Cell Repair Processes Following Organic Dust Exposure,” American Journal of Physiology‐Lung Cellular and Molecular Physiology 314 (2018): L421–L431. 29097425 10.1152/ajplung.00273.2017 PMC5900355

[36] T. M. Nordgren , C. D. Bauer , A. J. Heires , et al., “Maresin‐1 Reduces Airway Inflammation Associated With Acute and Repetitive Exposures to Organic Dust,” Translational Research 166 (2015): 57–69. 25655838 10.1016/j.trsl.2015.01.001 PMC4458456

[37] A. Ulu , S. Sveiven , A. Bilg , et al., “IL‐22 Regulates Inflammatory Responses to Agricultural Dust‐Induced Airway Inflammation,” Toxicology and Applied Pharmacology 446 (2022): 116044. 35525330 10.1016/j.taap.2022.116044 PMC9133182

[38] A. Ulu , A. Burr , A. J. Heires , et al., “A High Docosahexaenoic Acid Diet Alters Lung Inflammation and Recovery Following Repetitive Exposure to Aqueous Organic Dust Extracts,” Journal of Nutritional Biochemistry 97 (2021): 108797, 10.1016/j.jnutbio.2021.108797. 34126202 PMC8725620

[39] S. Carnevale , I. Di Ceglie , G. Grieco , A. Rigatelli , E. Bonavita , and S. Jaillon , “Neutrophil Diversity in Inflammation and Cancer,” Frontiers in Immunology 14 (2023): 1180810, 10.3389/fimmu.2023.1180810. 37180120 PMC10169606

[40] M. Evrard , I. W. H. Kwok , S. Z. Chong , et al., “Developmental Analysis of Bone Marrow Neutrophils Reveals Populations Specialized in Expansion, Trafficking, and Effector Functions,” Immunity 48 (2018): 364–379.e8. 29466759 10.1016/j.immuni.2018.02.002

[41] J. A. Poole , G. P. Dooley , R. Saito , et al., “Muramic Acid, Endotoxin, 3‐Hydroxy Fatty Acids, and Ergosterol Content Explain Monocyte and Epithelial Cell Inflammatory Responses to Agricultural Dusts,” Journal of Toxicology and Environmental Health, Part A 73 (2010): 684–700. 20391112 10.1080/15287390903578539 PMC2856089

[42] J. A. Poole , T. A. Wyatt , P. J. Oldenburg , et al., “Intranasal Organic Dust Exposure‐Induced Airway Adaptation Response Marked by Persistent Lung Inflammation and Pathology in Mice,” American Journal of Physiology‐Lung Cellular and Molecular Physiology 296 (2009): L1085–L1095. 19395665 10.1152/ajplung.90622.2008 PMC2692812

[43] R. Grieshaber‐Bouyer and P. A. Nigrovic , “Neutrophil Heterogeneity as Therapeutic Opportunity in Immune‐Mediated Disease,” Frontiers in Immunology 10 (2019): 346. 30886615 10.3389/fimmu.2019.00346 PMC6409342

[44] J. M. Adrover , J. A. Nicolás‐Ávila , and A. Hidalgo , “Aging: A Temporal Dimension for Neutrophils,” Trends in Immunology 37 (2016): 334–345. 27083489 10.1016/j.it.2016.03.005

[45] E. C. Dominguez , A. J. Heires , J. Pavlik , et al., “A High Docosahexaenoic Acid Diet Alters the Lung Inflammatory Response to Acute Dust Exposure,” Nutrients 12 (2020): 2334. 10.3390/nu12082334. 32759853 PMC7468878

[46] E. G. G. Sprenkeler , A. T. J. Tool , S. S. V. Henriet , R. van Bruggen , and T. W. Kuijpers , “Formation of Neutrophil Extracellular Traps Requires Actin Cytoskeleton Rearrangements,” Blood 139 (2022): 3166–3180. 35030250 10.1182/blood.2021013565

[47] C. Jones , A. La Flamme , P. Larsen , and K. Hally , “CPHEN‐017: Comprehensive Phenotyping of Neutrophil Extracellular Traps (NETs) on Peripheral Human Neutrophils,” Cytometry, Part A 105 (2024): 639–652. 10.1002/cyto.a.24851 38867433

[48] L. M. Silva , N. Moutsopoulos , T. H. Bugge , and A. Doyle , “Live Imaging and Quantification of Neutrophil Extracellular Trap Formation,” Current Protocols 1 (2021): e157. 34260822 10.1002/cpz1.157 PMC8288501

[49] M. Stoimenou , G. Tzoros , P. Skendros , et al., “Methods for the Assessment of NET Formation: From Neutrophil Biology to Translational Research,” International Journal of Molecular Sciences 23 (2022): 15823. 36555464 10.3390/ijms232415823 PMC9781911

[50] V. Brinkmann , C. Goosmann , L. I. Kühn , and A. Zychlinsky , “Automatic Quantification of In Vitro NET Formation,” Frontiers in Immunology 3 (2012): 413. 23316198 10.3389/fimmu.2012.00413 PMC3540390

[51] S. De Alessandris , G. J. Ferguson , A. J. Dodd , et al., “Neutrophil GM‐CSF Receptor Dynamics in Acute Lung Injury,” Journal of Leukocyte Biology 105 (2019): 1183–1194. 30942918 10.1002/JLB.3MA0918-347R PMC6850700

[52] A. Mulvanny , N. Jackson , C. Pattwell , et al., “The Dose Response of Inhaled LPS Challenge in Healthy Subjects,” European Journal of Inflammation 16 (2018): 205873921878482. 10.1177/2058739218784820.

[53] H. Wang , C. Aloe , N. Wilson , and S. Bozinovski , “G‐CSFR Antagonism Reduces Neutrophilic Inflammation During Pneumococcal and Influenza Respiratory Infections Without Compromising Clearance,” Scientific Reports 9 (2019): 17732. 31776393 10.1038/s41598-019-54053-w PMC6881371

[54] S. Martinelli , M. Urosevic , A. Daryadel , et al., “Induction of Genes Mediating Interferon‐Dependent Extracellular Trap Formation During Neutrophil Differentiation,” Journal of Biological Chemistry 279 (2004): 44123–44132. 15302890 10.1074/jbc.M405883200

[55] B. Amulic , S. L. Knackstedt , U. Abu Abed , et al., “Cell‐Cycle Proteins Control Production of Neutrophil Extracellular Traps,” Developmental Cell 43 (2017): 449–462.e5. 29103955 10.1016/j.devcel.2017.10.013

[56] J. Qu , J. Jin , M. Zhang , and L. G. Ng , “Neutrophil Diversity and Plasticity: Implications for Organ Transplantation,” Cellular & Molecular Immunology 20 (2023): 993–1001. 37386174 10.1038/s41423-023-01058-1 PMC10468536

[57] K. Ganesh and M. B. Joshi , “Neutrophil Sub‐Types in Maintaining Immune Homeostasis During Steady State, Infections and Sterile Inflammation,” Inflammation Research 72 (2023): 1175–1192. 37212866 10.1007/s00011-023-01737-9 PMC10201050

[58] J. M. Adrover , C. del Fresno , G. Crainiciuc , et al., “A Neutrophil Timer Coordinates Immune Defense and Vascular Protection,” Immunity 50 (2019): 390–402.e10. 30709741 10.1016/j.immuni.2019.01.002

[59] G. Drifte , I. Dunn‐Siegrist , P. Tissières , and J. Pugin , “Innate Immune Functions of Immature Neutrophils in Patients With Sepsis and Severe Systemic Inflammatory Response Syndrome*,” Critical Care Medicine 41 (2013): 820–832. 23348516 10.1097/CCM.0b013e318274647d

[60] N. Krishnamoorthy , D. N. Douda , T. R. Brüggemann , et al., “Neutrophil Cytoplasts Induce TH17 Differentiation and Skew Inflammation Toward Neutrophilia in Severe Asthma,” Science Immunology 3 (2018): eaao4747. 10.1126/sciimmunol.aao4747. 30076281 PMC6320225

[61] D. Zhang , G. Chen , D. Manwani , et al., “Neutrophil Ageing Is Regulated by the Microbiome,” Nature 525 (2015): 528–532. 26374999 10.1038/nature15367 PMC4712631

[62] H. I. Warheit‐Niemi , G. P. Huizinga , S. J. Edwards , et al., “Fibrotic Lung Disease Alters Neutrophil Trafficking and Promotes Neutrophil Elastase and Extracellular Trap Release,” Immunohorizons 6 (2022): 817–834. 36534439 10.4049/immunohorizons.2200083 PMC10542701

[63] R. Lokwani , P. Wark , K. Baines , et al., Circulatory neutrophils in COPD feature downregulated CD62L expression in comparison with asthma and healthy participants European Respiratory Society (Allergy and immunology).2019,PA4384.

[64] E. Fortunati , K. M. Kazemier , J. C. Grutters , L. Koenderman , and V. J. M. M. Van den Bosch , “Human Neutrophils Switch to an Activated Phenotype After Homing to the Lung Irrespective of Inflammatory Disease,” Clinical and Experimental Immunology 155 (2009): 559–566. 19077082 10.1111/j.1365-2249.2008.03791.x PMC2669533

[65] R. Lokwani , P. A. Wark , K. J. Baines , M. Fricker , D. Barker , and J. L. Simpson , “Blood Neutrophils In,” International Journal of Chronic Obstructive Pulmonary Disease 14 (2019): 2517–2525. 31814717 10.2147/COPD.S222486 PMC6863133

[66] A. E. Williams and R. C. Chambers , “The Mercurial Nature of Neutrophils: Still an Enigma in ARDS?,” American Journal of Physiology‐Lung Cellular and Molecular Physiology 306 (2014): L217–L230. 24318116 10.1152/ajplung.00311.2013 PMC3920201

[67] P. Rees , H. D. Summers , A. Filby , A. E. Carpenter , and M. Doan , “Imaging Flow Cytometry,” Nature Reviews Methods Primers 2 (2022): 86. 10.1038/s43586-022-00167-x PMC10468826 37655209

