# Supplementary material for: Spectral Flow Cytometry Method for Immunophenotyping Neutrophil Activation and NETs in an Acute Dust Exposure Model
Source: Immun Inflamm Dis. 2026 Jun 30;14(6):e70482. doi: 10.1002/iid3.70482 (PMC13316450; doi:10.1002/iid3.70482)
Supplement: Supplementary file 1 — Figure S1: Median Fluorescent Intensity (MFI) of CXCR2 and CXCR4 in banded and mature neutrophils in bone marrow from PBS‐expose mice. Mann Whitney‐U Test, ****p < 0.0001. [file IID3-14-e70482-s002.docx]

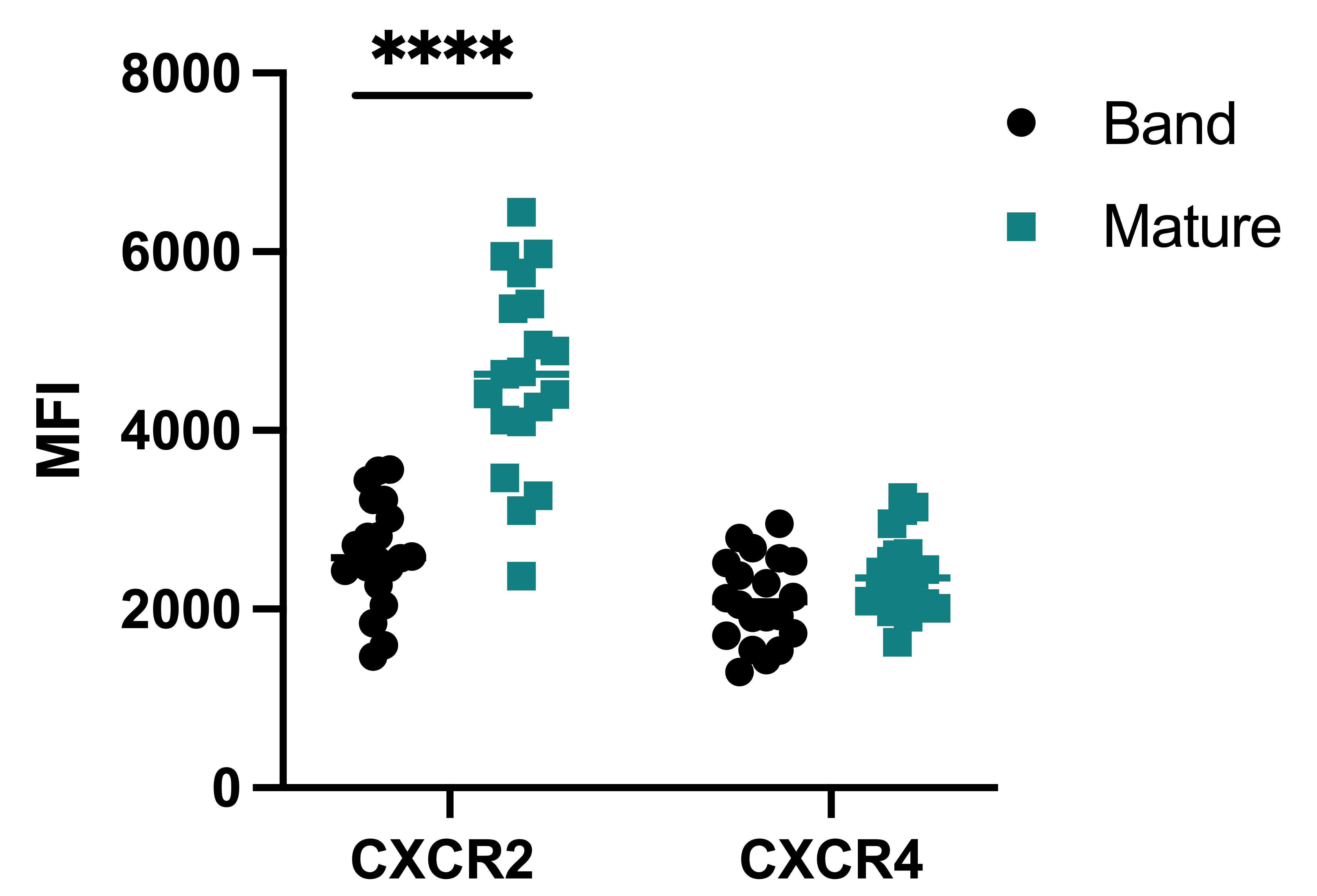


**Supplementary Figure 1.** Median Fluorescent Intensity (MFI) of CXCR2 and CXCR4 in banded and mature neutrophils in bone marrow from PBS-expose mice. Mann Whitney-U Test, ****p<0.0001.
