# Supplementary material for: Spectral Flow Cytometry Method for Immunophenotyping Neutrophil Activation and NETs in an Acute Dust Exposure Model
Source: Immun Inflamm Dis. 2026 Jun 30;14(6):e70482. doi: 10.1002/iid3.70482 (PMC13316450; doi:10.1002/iid3.70482)
Supplement: Supplementary file 2 — Figure S2: Cell expressing CitH3, MPO, or co‐expressing CitH3 and MPO were significantly increased following ODE. (A) Representative images of CitH3 and MPO immune‐fluorescence staining in PBS‐ and ODE‐exposed mice (200X magnification, scale bar 100 µM). (B) Quantification of 5 images from PBS‐ and ODE‐exposed mice (n = 4). Each dot represents total cell numbers expressing the proteins from a single image. Asterisks denote a two‐tail p value ≤ 0.05 as computed by unpaired Mann Whitney‐U test. [file IID3-14-e70482-s005.docx]

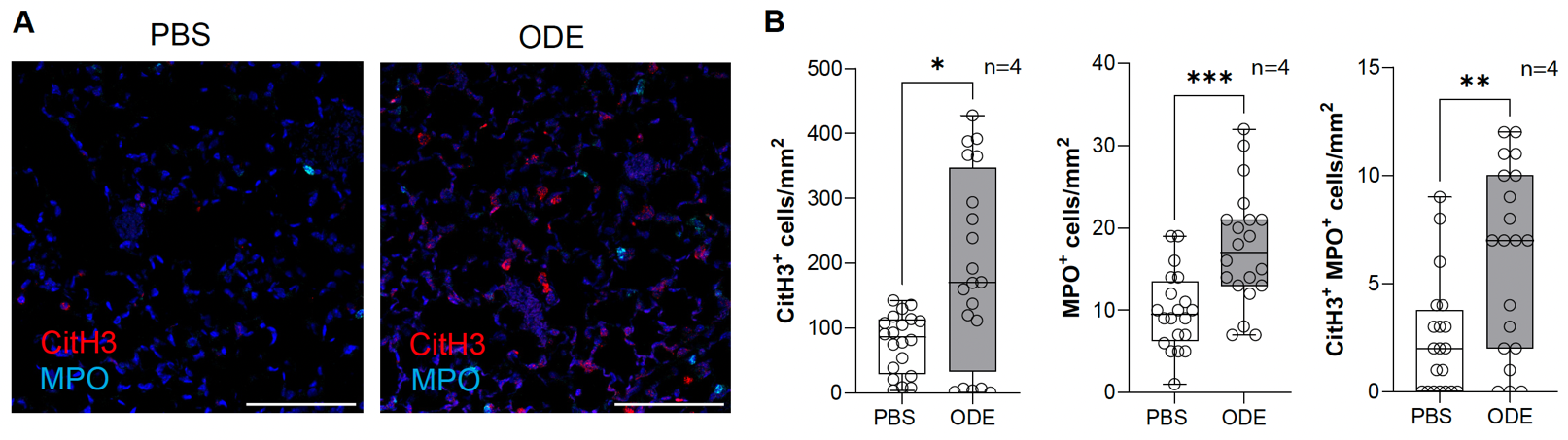
**Supplementary Figure 2**: Cell expressing CitH3, MPO, or co-expressing CitH3 and MPO were significantly increased following ODE. **A.)** Representative images of CitH3 and MPO immune-fluorescence staining in PBS- and ODE-exposed mice (200X magnification, scale bar 100µM). **B.)** Quantification of 5 images from PBS- and ODE-exposed mice (n=4). Each dot represents total cell numbers expressing the proteins from a single image. Asterisks denote a two-tail p value ≤ 0.05 as computed by unpaired Man-Whitney test.
