# Supplementary material for: Spectral Flow Cytometry Method for Immunophenotyping Neutrophil Activation and NETs in an Acute Dust Exposure Model
Source: Immun Inflamm Dis. 2026 Jun 30;14(6):e70482. doi: 10.1002/iid3.70482 (PMC13316450; doi:10.1002/iid3.70482)
Supplement: Supplementary file 3 — Figure S3: (A) MPO+ band and mature neutrophil counts and (B) percentages in the lung samples exposed to PBS or ODE. Mann Whitney‐U Test *p < 0.05, ****p < 0.0001, ns = non‐significant. [file IID3-14-e70482-s001.docx]

**Supplementary Figure 3: A.)** MPO^+^ band and mature neutrophil counts and **B.)** percentages in the lung samples exposed to PBS or ODE. Mann Whitney-U Test *p<0.05, ****p<0.0001, ns = non-significant.
