# Supplementary material for: Spectral Flow Cytometry Method for Immunophenotyping Neutrophil Activation and NETs in an Acute Dust Exposure Model
Source: Immun Inflamm Dis. 2026 Jun 30;14(6):e70482. doi: 10.1002/iid3.70482 (PMC13316450; doi:10.1002/iid3.70482)
Supplement: Supplementary file 4 — Figure S4: Representative gating strategy for fluorescent minus one (FMO) samples utilized for gating placement for NET formation markers MPO and CitH3 in the lung and BALF in mature and band neutrophil populations6. [file IID3-14-e70482-s004.docx]

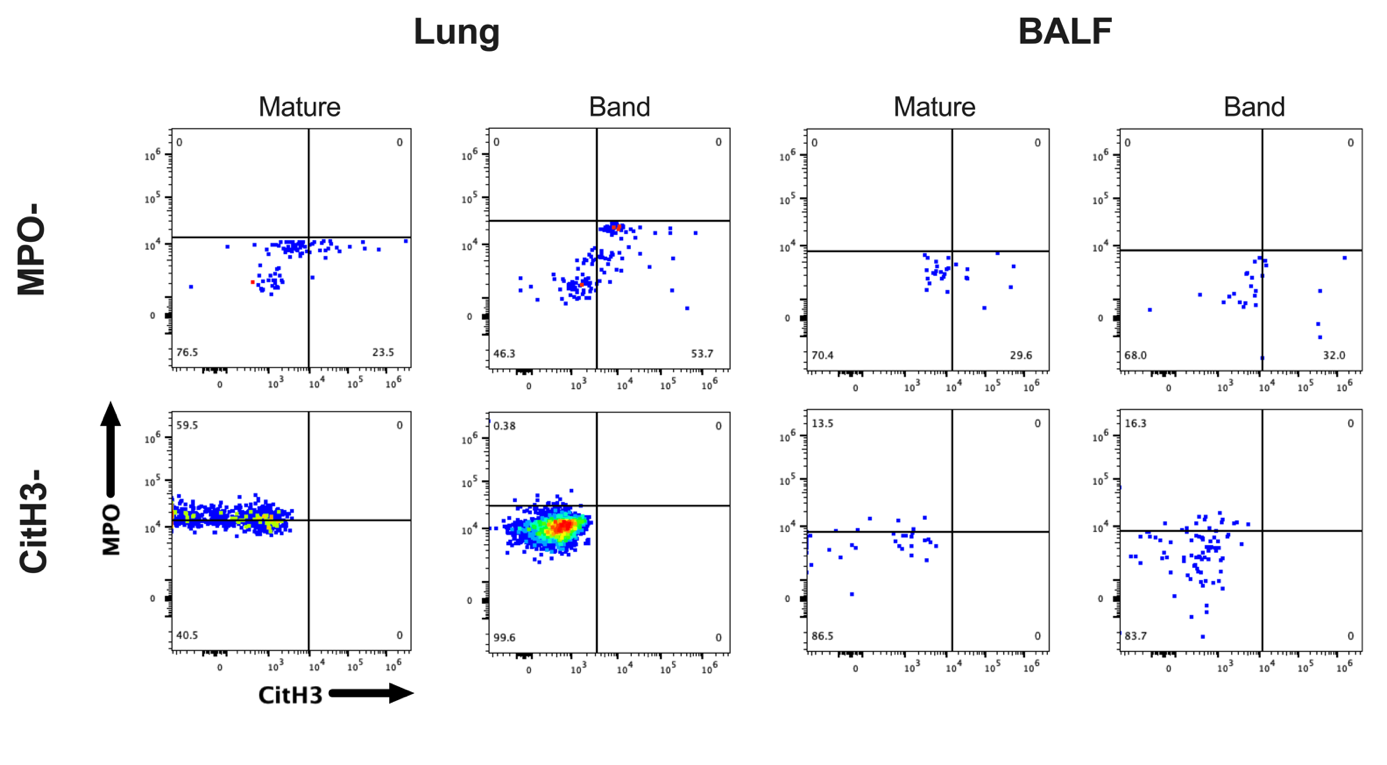


**Supplementary Figure 4**: Representative gating strategy for fluorescent minus one (FMO) samples utilized for gating placement for NET formation markers MPO and CitH3 in the lung and BALF in mature and band neutrophil populations.
