# Supplementary material for: Spectral Flow Cytometry Method for Immunophenotyping Neutrophil Activation and NETs in an Acute Dust Exposure Model
Source: Immun Inflamm Dis. 2026 Jun 30;14(6):e70482. doi: 10.1002/iid3.70482 (PMC13316450; doi:10.1002/iid3.70482)
Supplement: Supplementary file 5 — Table S1: Fluorescently conjugated antibodies used for flow cytometry. Panel denotes extracellular (ex) and intracellular (in) location of markers. [file IID3-14-e70482-s003.docx]

| **Marker** | **Fluor** | **Manufacturer** | **Dilution** | **Concentration** | **Panel** |
| --- | --- | --- | --- | --- | --- |
| **Ly6G** | PE-Dazzle 594 | Biolegend, Cat# 127648, Clone:1A8 | 1:400 | 0.5 ug/mL | ex |
| **CD34** | BV605 | BD Biosciences, Cat# 17222185, Clone RAM34 | 1:800 | 1.25 ug/mL | ex |
| **CD117** | BV650 | Biolegend, Cat# 563399, Clone:2B8 | 1:400 | 0.5 ug/mL | ex |
| **CD62L** | BV711 | Biolegend, Cat# 104445, Clone: MEL-14 | 1:800 | 0.5 ug/mL | ex |
| **CXCR2** | BV786 | BD Biosciences, Cat# 747811, Clone: V48-2310 | 1:400 | 0.5 ug/mL | ex |
| **CXCR4** | PerCP-eFlour 710 | Thermofisher Scientific Cat# 46-9991-82, Clone: 2B11 | 1:400 | 0.5 ug/mL | ex |
| **MPO** | PE | Abcam, Cat# AF3667, Thermofisher Scientific Cat# PA1-29953 | 1:100 1°, 1:400 2° | 2 ug/mL, 2.58 ug/mL | in |
| **CitH3** | APC | Abcam, Cat# ab281584, Thermofisher Scientific Cat# A10931 | 1:100 1°, 1:400 2° | 5 ug/mL, 2.5 ug/mL | in |
| **CD11b** | AF700 | Biolegend, Cat# 101222, Clone: M1/70 | 1:800 | 0.625 ug/mL | ex |
| **NK1.1** | FITC | Biolegend, San Diego, CA. Cat# 156507, Clone: S17016D | 1:1600 | 0.625 ug/mL | ex |
| **CD3** |  | Biolegend, San Diego, CA. Cat# 100305, Clone: [145-2C11](https://www.biolegend.com/nl-nl/search-results?Clone=RA3-6B2) |  | 0.313 ug/mL | ex |
| **B220** |  | Biolegend, San Diego, CA. Cat# 103205, Clone: [RA3-6B2](https://www.biolegend.com/nl-nl/search-results?Clone=RA3-6B2) |  | 0.313 ug/mL | ex |
| **CD19** |  | Biolegend, San Diego, CA. Cat# 152403, Clone: [1D3/CD19](https://www.biolegend.com/nl-nl/search-results?Clone=RA3-6B2) |  | 0.313 ug/mL | ex |
| **Ter119** | AF488 | Biolegend, San Diego, CA. Cat# 116215, Clone: TER119 | 1:1600 | 0.313 ug/mL | ex |
| **DNA** | Hoechst 33342 | Thermofisher Scientific, Cat# R37165 | 2 drops/mL | 2 drops/mL | ex |
| **Viability** | Ghost Red 780 | Tonbo Biosciences, Cat#13-0865-T100 | 1:5000 | 0.2 ul/mL | ex |

**Supplementary Table 1:** Fluorescently conjugated antibodies used for flow cytometry. Panel denotes extracellular (ex) and intracellular (in) location of markers.
